# Virtual Insanity: Perspectives from a Political Digital Ethnographer of Young Adults Using Social Media for Mental Health

**DOI:** 10.1192/j.eurpsy.2024.136

**Published:** 2024-08-27

**Authors:** A. Bailie

**Affiliations:** Politics and International Relations, University of York, York, United Kingdom

## Abstract

**Abstract:**

To contribute to this debate I offer perspectives from my PhD research which critically examines the contemporary U.K politics of mental health and illness amongst young adults via social media. My work examines the way in which social media, like Instagram and Tiktok allows young adults to explore, express and share their selfhood and identity around ideas of mental health and illness through videos, posts and online interactions. Through this work I have engaged with digital services, psychologists and medical professionals on the subject of using technology for the treatment, engagement of and knowledge of mental health and illness. I have additionally engaged with some work on the role of the Metaverse for treating mental illness, and how this could work, but also the limitations of virtual spaces. Exploring debates in digital sociology adds evidence to these arguments and can support the understanding of the political ramifications of using technologies in the clinical space. Arguing that these new developments in language and social practices around mental health and illness via social media need to be further explored, acknowledged and addressed in social science and this can be supported by work in the field of psychiatry. Overall, my contribution to the debate will be to offer political and digital social perspectives on the use of technology and highlight some of the biases and drawbacks of utilising AI to treat mental health and illness.

**Disclosure of Interest:**

None Declared

